# Cleavage and Sub-Cellular Redistribution of Nuclear Pore Protein 98 by Coxsackievirus B3 Protease 2A Impairs Cardioprotection

**DOI:** 10.3389/fcimb.2019.00265

**Published:** 2019-07-24

**Authors:** Paul J. Hanson, Al Rohet Hossain, Ye Qiu, Huifang M. Zhang, Guangze Zhao, Cheng Li, Veena Lin, Saheedat Sulaimon, Marli Vlok, Gabriel Fung, Victoria H. Chen, Eric Jan, Bruce M. McManus, David J. Granville, Decheng Yang

**Affiliations:** ^1^Department of Pathology and Laboratory Medicine, University of British Columbia, Vancouver, BC, Canada; ^2^UBC Centre for Heart Lung Innovation, St. Paul's Hospital, Vancouver, BC, Canada; ^3^Jefferson College of Population Health, Thomas Jefferson University, Philadelphia, PA, United States; ^4^Department of Biochemistry and Molecular Biology, University of British Columbia, Vancouver, BC, Canada; ^5^Faculty of Medicine, University of British Columbia, Vancouver, BC, Canada

**Keywords:** CVB3, NUPs, NUP98, NRG1, ERBB4, PSEN1, protease, myocarditis

## Abstract

Myocarditis, inflammation of the heart muscle, affects all demographics and is a major cause of sudden and unexpected death in young people. It is most commonly caused by viral infections of the heart, with coxsackievirus B3 (CVB3) being among the most prevalent pathogens. To understand the molecular pathogenesis of CVB3 infection and provide strategies for developing treatments, we examined the role of a key nuclear pore protein 98 (NUP98) in the setting of viral myocarditis. NUP98 was cleaved as early as 2 h post-CVB3 infection. This cleavage was further verified through both the ectopic expression of viral proteases and *in vitro* using purified recombinant CVB3 proteases (2A and 3C), which demonstrated that CVB3 2A but not 3C is responsible for this cleavage. By immunostaining and confocal imaging, we observed that cleavage resulted in the redistribution of NUP98 to punctate structures in the cytoplasm. Targeted siRNA knockdown of NUP98 during infection further increased viral protein expression and viral titer, and reduced cell viability, suggesting a potential antiviral role of NUP98. Moreover, we discovered that expression levels of neuregulin-1 (NRG1), a cardioprotective gene, and presenilin-1 (PSEN1), a cellular protease processing the tyrosine kinase receptor ERBB4 of NRG1, were reliant upon NUP98 and were downregulated during CVB3 infection. In addition, expression of these NUP98 target genes in myocardium tissue not only occurred at an earlier phase of infection, but also appeared in areas away from the initial inflammatory regions. Collectively, CVB3-induced cleavage of NUP98 and subsequent impairment of the cardioprotective NRG1-ERBB4/PSEN1 signaling cascade may contribute to increased myocardial damage in the context of CVB3-induced myocarditis. To our knowledge, this is the first study to demonstrate the link between NUP98 and the NRG1 signaling pathway in viral myocarditis.

## Introduction

Myocarditis, defined as inflammation of the myocardium, encompasses a spectrum of conditions causing considerable morbidity and mortality. There are considerable gaps in our knowledge relating to the molecular pathogenesis of the disease (Feldman and McNamara, 2000). Although myocarditis affects all demographics, it is among the leading causes of sudden and unexpected death in children and young adults, with males being preferentially affected as compared to females. Long-term manifestations of myocarditis include arrhythmia, chronic active myocarditis and dilated cardiomyopathy (DCM), all of which may result in heart failure. Viruses are the major causal agents of infectious myocarditis (Feldman and McNamara, 2000). Coxsackievirus B3 (CVB3) is among the most prevalent cardiotropic pathogens of viral myocarditis (Yajima and Knowlton, 2009) and has been implicated in ~20% of acute onset heart failure and DCM (Baboonian and Treasure, 1997; Baboonian et al., 1997). At the present, treatment for myocarditis is mostly supportive and there is no widely approved specific therapy or vaccine for CVB3 (Abelmann, 1971; McManus et al., 1988; Schultheiss et al., 2011). For DCM patients, the only definitive treatment thus far is mechanical assist devices and/or heart transplantation.

CVB3 is a positive, single-stranded RNA virus in the genus enterovirus of family *Picornaviridae*; its genome encodes a single long open reading frame flanked by the 5′ and 3′ untranslated regions (UTR). Unlike the cellular mRNAs that link to a m^7^G(5′)ppp(5′)N cap structure at the 5′ terminus, the CVB3 genome is attached with a small viral peptide, VPg and its 5′UTR harbors an internal ribosome entry site (IRES), which drives the translation of viral RNA via a cap-independent mechanism (Liu et al., 1999). The translated long polyprotein can be processed by viral proteases 2A and 3C into four structural (VP1-4) and seven non-structural proteins, including proteases 2A and 3C as well as other proteins used for viral RNA replication and infectious particle assembly (Sin et al., 2015; Fung et al., 2016). Enteroviral proteases have been well-documented in their roles to suppress multiple host cell activities via cleavage of cellular proteins, For example, enterovirus proteases can cleave eukaryotic transcription factors and activators (Yalamanchili et al., 1997; Castello et al., 2011). Protease 2A can cleave eukaryotic initiation factors 4GI (eIF4GI) (Lamphear et al., 1995; Lamphear and Rhoads, 1996; Chau et al., 2007) and eIF4GII (Gradi et al., 1998; Svitkin et al., 1999) shutting down cap-dependent translation. In addition, some enterovirus proteases can also cleave cellular structure proteins, such as dystrophin and nucleopore proteins (NUPs) that are involved in maintaining cellular integrity and functions (Xiong et al., 2002; Park et al., 2008).

All positive-polarity single-stranded RNA viruses are cytoplasmic viruses, which recruit and rearrange host cellular membranes to form vesicles for viral replication (Schwartz et al., 2004). For example, Hepatitis C virus (HCV) induces sub-cellular redistribution of NUPs to the cytoplasm, where they accumulate at sites enriched for HCV proteins and viral assembly (Neufeldt et al., 2013). Furthermore, the depletion of NUPs via siRNA knockdown inhibits HCV replication. In addition, poliovirus 2A protease can target NUP98, NUP62 and NUP153 for cleavage during the course of infection (Park et al., 2008), resulting in the cellular relocalization of NUPs. This cleavage benefits poliovirus replication (Fitzgerald et al., 2013). However, with respect to CVB3, a close relative of poliovirus that also replicates in membrane vesicles within the cytoplasm but causes different diseases, whether proteases can cleave NUPs in order to augment pathogenesis is unknown.

The nuclear pore complex is a 125 MDa complex made up of over 40 different proteins (Liang et al., 2013; Cautain et al., 2015). NUPs were traditionally thought to be mere “gatekeepers” of the nucleus, regulating the translocation of nuclear resident proteins and RNA in and out of the nucleus. In recent years, multiple, dynamic functions, such as transcriptional activation or repression, have been described, whereby NUPs migrate from the nuclear periphery and interact directly with chromatin or as direct transcription factors. One NUP in particular, NUP98, performs both nuclear gate-keeping functions, as well as acting as an immune-responsive transcription factor (Panda et al., 2014, 2015). This dual function of NUP98 has been demonstrated during human development and in various pathological conditions (Xu and Powers, 2009; Franks and Hetzer, 2013; Lussignol et al., 2016). During human heart development, NUP98 has been shown to be a transcription factor for neuregulin-1 (NRG1) (Liang et al., 2013), a protein which plays an integral role during development in trabeculation and myocyte proliferation resulting in the thickening and proper functioning of the ventricles. The interaction between NRG1 and its tyrosine kinase receptor, epidermal growth factor ERBB4, has also been shown to be beneficial in promoting cardioprotective effects post-ischemia (Wang et al., 2018) as well as being involved in host cell response to infection (Ho et al., 2017). Therefore, NRG-1 and its receptor ERBB4 may be of central importance to the recovery from acute and chronic CVB3 induced myocarditis. Additionally, Presenilin-1 (PSEN1), a cellular protease and component of the γ-secretase complex, has been demonstrated to cleave ERBB4 and translocate with it to the nucleus in cells undergoing apoptosis (Vidal et al., 2005). Indeed, NRG-1, PSEN1 and ERBB4 dysfunction have been described as key contributors in the pathogenesis of cancer, cardiovascular disease, and neurological disorders (Woo et al., 2011; Cui et al., 2013; Vasti and Hertig, 2014). However, whether this finding can fill the knowledge gap in our understanding of molecular pathogenesis of CVB3-induced viral myocarditis has not been investigated.

In the present study, we sought to understand the role of NUP98 in the pathogenesis of viral myocarditis and its impact on the hypothetically cardioprotective NRG1-ERBB4/PSEN1 signaling axis. We demonstrate that NUP98 is targeted early during CVB3 infection for cleavage by viral protease 2A. This cleavage leads to redistribution of NUP98 from the nucleus to the cytoplasm, where it forms punctuate structures. Further, knockdown of NUP98 resulted in dramatic reduction of its transcriptional targets leading to impaired cardioprotection, increased viral titer and decreased cell viability during infection. Additionally, Nup98, Nrg1, and erbB4 were all upregulated in the murine myocardium during acute/early phase of CVB3 infection, and Nrg-1 and erbB4 were downregulated at a later phase of infection, suggesting a cardioprotective role of this signal cascade in cellular response to infection.

## Materials and Methods

### Virus, Cells, Plasmids, and Animals

CVB3 (CG) strain was obtained from Dr. Charles Gauntt (University of Texas Health Science Center, San Antonio, TX) and propagated in HeLa cells (ATCC). Virus stock was isolated from cells by three freeze-thaw cycles followed by centrifugation to remove cell debris, and stored at −80°C. Virus stock titer was determined by plaque assay as described in a later section. HeLa cells were grown and maintained in Dulbecco's modified Eagle's medium (DMEM) supplemented with 10% fetal bovine serum (FBS) (Clontech, Palo Alto, CA), 100 μg/ml penicillin, 100 μg/ml streptomycin, and 2 mM glutamine. Plasmid construction of pIRES-2A and pIRES-3C was previously described (Fung et al., 2013). NUP98-GFP plasmid was a generous gift from the Fahrenkrog laboratory at the Université Libre de Bruxelles, Belgium. The animal experiments were carried out in accordance with the recommendations in the Guide to the Care and Use of Experimental Animals—Canadian Council on Animal Care. All of the animal protocols were approved by the Animal Care Committee, University of British Columbia (protocol number: A16-00593). Four-weeks old male A/J mice were purchased from Jackson Laboratories (Maine, USA). Ten mice in two groups were infected with 10^4^ plaque forming units (PFU) of CVB3 or sham infected with phosphate-buffered saline (PBS) (Sigma, St. Louis, MO) by intraperitoneal inoculation. Mice were monitored and sacrificed at the corresponding day post-infection (dpi). Seven dpi represents the acute phase of infection and 40 dpi represents the chronic phase. Hearts were harvested and a transverse section through the ventricles was fixed for at least 24 h in 10% phosphate-buffered formalin. The tissues were embedded in paraffin blocks and 4-μm tissue sections were stained with hematoxylin and eosin (H&E) for assessment of myocardial inflammation or via immunohistochemistry for determining protein expression (see section below).

### Transfection and Virus Infection

HeLa cells growing in 6-well plates at 70–80% confluence were washed with PBS and overlaid for 48 h with transfection complex containing 2 μg of plasmid DNA and 10 μl of lipofectamine 2000 (Invitrogen) reagent per well. siRNA specific to human NUP98 or scrambled siRNA (Santa Cruz) was transfected at a final concentration of 10 μM for 48 h using oligofectamine reagent. The transfection medium was then replaced with DMEM containing 10% FBS and the incubation was continued for 24 h prior to viral infection, or 48 h prior to harvesting. For viral infection, cells were washed with PBS and infected in 500 μl of serum-free DMEM at a multiplicity of infection (MOI) of 10 or sham-infected with PBS for 3, 5, or 7 h.

### Western Blot Analysis

All Western blot analyses were conducted using previously described standard protocols (Felker et al., 1999). Mouse heart tissue was sectioned from the apical tissue and homogenized in 1% Triton X-100 MOSLB lysis buffer containing protease inhibitor cocktail. Tissue lysates were centrifuged at 4°C, 13,000 × g for 20 min. The supernatants were collected and protein concentrations were measured using the Bradford Assay (Bio-Rad Laboratories, Mississauga, ON). Cells were washed twice in cold PBS after transfection or infection at different time points mentioned above. Cells were lysed in lysis buffer (0.025 M Tris-HCl, pH 8.0, 137 mM NaCl, 10% glycerol, 1 mM EDTA, 1 mM EGTA, 1%Triton X-100 and protease inhibitor cocktail) on ice for 20 min. Supernatants containing proteins were isolated by centrifugation at 13,000 × g at 4°C for 15 min. Proteins were separated by 10% SDS-polyacrylamide gel electrophoresis (SDS-PAGE) and transferred onto nitrocellulose membranes (Amersham). Membranes were blocked in 5% skim milk in Tris-buffered saline 10% tween (TBST) buffer for 1 h and subsequently incubated with one of the following primary antibodies against NUP98 (Cell signaling), Neuregulin-1 and ERBB4 (Thermo Fisher), PSEN1 (Abcam), VP1 (Dako), and β-actin (Santa Cruz) at 4°C overnight. Membranes were subsequently washed in TBST three times for 10 min each, followed by incubation with the appropriate secondary antibody (goat anti-mouse or donkey anti-rabbit) conjugated to horseradish peroxidase (Amersham) and visualized using the enhanced chemiluminescence method as per the manufacturer's instructions (Amersham). Protein expression was quantified by densitometry using the NIH ImageJ software package (http://imagej.nih.gov/ij/index.html) and normalized to GAPDH or β-actin as indicated based upon an *n* of 3 for each experiment and depicted graphically next to each blot in the figure. Sham values were normalized to 1.00.

### *In vitro* Protein Cleavage Assay

Recombinant CVB3 wild-type and catalytically inactive mutant 2A and 3C proteases were purified as described previously (Jagdeo et al., 2018). For cleavage assays, 25 μg of whole-cell lysates prepared from HeLa cells was incubated with CVB3 2A (5 ng/μl) or CVB3 3C (100 ng/μl) at 37°C for 4 h in reaction buffer [20 mM Hepes (pH7.4), 150 mM KOAc, and 1 mM DTT] (Park et al., 2015). After this incubation, samples were analyzed by Western blot analysis as described above.

### Immunocytochemistry

HeLa cells proliferating on glass coverslips in a 6-well plate at ~70% confluence were transfected with pIRES-vector, pIRES-2A or pIRES-3C. At 48 h post-transfection, cells were stained as described previously (Felker et al., 2000). Briefly, cells were fixed with 4% paraformaldehyde, permeabilized in methanol/acetone (50:50) at −20°C for 20 min and stained with an anti-NUP98 primary antibody (Cell signaling). Slides were washed and stained with a goat anti-rabbit IgG (H + L) labeled with ALEXA Fluor 488 (Invitrogen). Nuclei were stained with 4′,6′-diamidine-2′-phenylindole dihydrochloride (DAPI) (Vector Laboratories). Cells were observed under a Leica SP2 AOBS confocal microscope. Mouse hearts were harvested from 4-weeks old CVB3-infected A/J mice and fixed in 10% formalin (Fisher) for at least 24 h. Hearts were then imbedded in paraffin and sectioned in 4 μM sections onto slides for staining. For evaluation of inflammation and damage, Hematoxylin and Eosin (H&E) staining was conducted by following the method described previously (Wang et al., 2011). For immunochemistry staining, sections were deparaffinized, rehydrated and subjected to citrate buffer (pH 6.0, Invitrogen) heat-induced antigen retrieval. Slides were washed in TBS and blocked for 30 min in TBS/5% BSA solution at room temperature. Slides were incubated overnight at 4°C with a primary antibody (NUP98, NRG-1, PSEN1, or ERBB4) diluted in TBS/1% BSA according to the manufacturer's suggestion. The following day, the slides were treated with Mach 4 polymer detection complex-alkaline phosphatase (Biocare Medical, USA) amplification step followed by Warp Chromogen Red (Biocare Medical) as the substrate. Hematoxylin was used to counterstain cell nuclei. Bright field imaging under the Nikon Eclipse E600 microscope (Nikon, El Segundo, USA) was digitally captured using a SpotFlex camera (Diagnostic Instruments, Sterling Heights, USA), with *n* = 3 mouse hearts for each antigen detected. Proportion of Positive Area (PPA) Image analysis was performed on the Aperio ImageScopeTM following analysis workflow recommended by the Aperio ImageScopeTM User's Guide (https://www.leicabiosystems.com/digital-pathology/manage/aperio-imagescope/) (Kalra et al., 2015). Tissues were traced to indicate area for image analysis, while artifacts, such as folded regions of tissue were removed from the analysis. PPA was calculated by adding the number of positive and number of strong positive counts, then dividing this sum by the total number of positive and negative counts as provided by image analysis results. Differential expression of Nup98, erbB4 and Nrg1 in sham infected vs. CVB3 infected murine myocardium at the indicated dpi (*n* = 3) were quantified using a fold change value. This value was calculated by dividing the number of positive and strong positive signal by the total number of positive signal and negative signal. The average PPA of three sham infected hearts was set to 1.00. Therefore, the fold change value is effectively an expression of protein expression normalized to sham infected heart tissue. Statistical analyses are described below.

### Viral Plaque Assay

Viral titers were determined as previously described (Yuan et al., 2009). Briefly, HeLa cells were seeded into 6-well plates (8 × 10^5^ cells/well) and incubated at 37°C for 20 h to a confluence of ~90%, then washed with PBS and overlaid with 500 μl of virus serially diluted in cell-culture medium. Viruses were obtained by centrifugation (4,000 × g) of freeze-thawed cell suspensions as described above. After a viral-adsorption period of 60 min at 37°C, the supernatant was removed, the cells overlaid with 2 ml of sterilized soft Bacto-agar-minimal essential medium, cultured at 37°C for 72 h, fixed with Carnoy's fixative for 30 min, and stained with 1% crystal violet. The plaques were counted and PFU per ml calculated.

### Cell Viability Assay

HeLa cell viability was quantified using a 3-(4-5-dimethylthiazol-2-yl)-5-(-3-carboxymethoxyphenyl)-2H-tetrazolium salt (MTS) assay kit following the manufacturer's instructions (Promega). Cells were transfected with siRNA specific to human NUP98 or scrambled siRNA. Transfected cells were infected with CVB3 at an MOI of 10 or sham-infected with PBS (see transfection and virus infection section above). Briefly, cells were incubated with MTS solution for 4 h. Absorbency of formazan was measured at 492 nm using an enzyme-linked immunosorbent assay (ELISA) plate reader with *n* ≥ 3 for each time point. Absorbency of sham infected/scrambled siRNA treated cells was defined as 100% viability (control) and the remaining data were converted to a percentage of the control.

### Statistical Analysis

Student's *t*-test was employed to analyze the data. Results are expressed as means ± standard deviation of three independent experiments. A *P-*value < 0.05 was considered statistically significant.

## Results

### NUP98 Is Cleaved by Viral Protease 2A but Not 3C During CVB3 Infection

**Figure 1:**
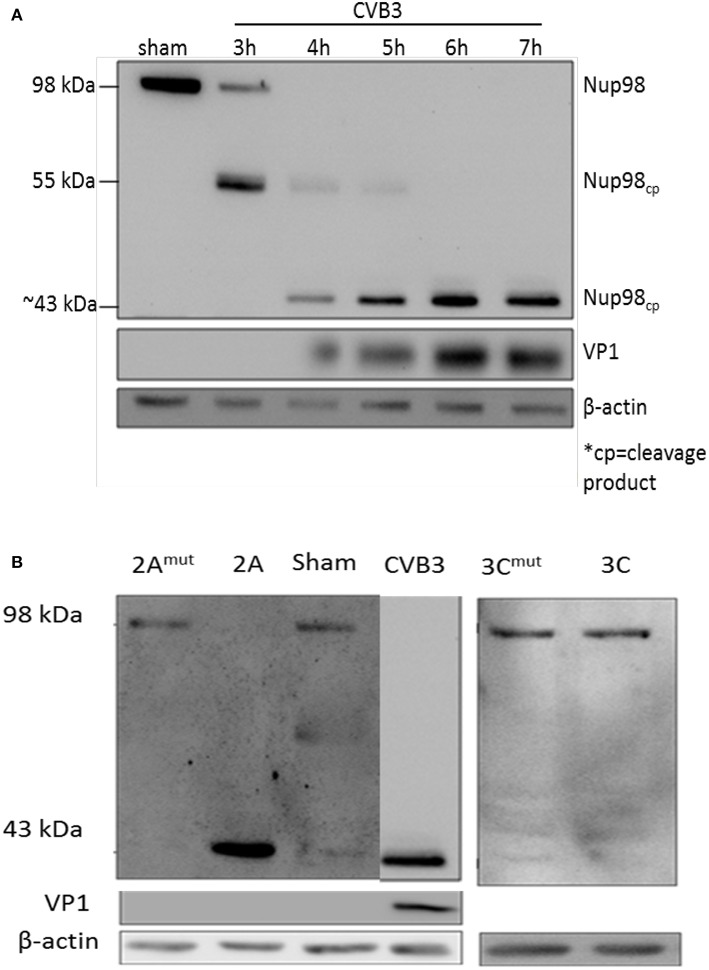
NUP98 is cleaved by viral protease 2A during CVB3 infection. **(A)** NUP98 cleavage in CVB3-infected HeLa cells. HeLa cells were infected with CVB3 or sham-infected with PBS. At the indicated time points post-infection (pi), cell lysates were harvested for western blot analysis of NUP98 cleavage products. VP1, a viral capsid protein, was used to represent viral replication. β-actin was used as a loading control. Molecular weight markers in kDa are indicated. **(B)** *In vitro* cleavage assay. Non-infected HeLa cells lysates were incubated overnight with purified recombinant viral protease 2A, 3C, 2A^mut^ , or 3C^mut^ (mut indicates mutated, inactive catalytic sites). Lysates were subjected to western blot analysis of NUP98 cleavage products. CVB3-infected cell lysate harvested at 7 h pi was used as a positive control. β-actin was used as a loading control.

To determine whether NUP98 is cleaved during CVB3 infection, HeLa cells were infected with CVB3 at a MOI of 10 or sham-infected with PBS. Sham-infected cells were collected corresponding to the collection of the final CVB3-infected time point (i.e., 8 hpi). Western blot analysis of HeLa cell proteins was conducted using an anti-NUP98 (targeting N-terminal to mid region of NUP98) antibody. Figure 1A demonstrates that a ~55 kDa cleavage fragment of NUP98 was generated by 3 h post-infection (hpi) from its 98 kDa proform. Further, a secondary ~43 kDa cleavage fragment of Nup98 was observed at 4 hpi. This ~43 kDa fragment increased in abundance throughout the duration of the infection (7 h). To further verify the viral protease-mediated cleavage, *in vitro* cleavage assay using purified recombinant CVB3 proteases 2A and 3C incubated with proteins extracted from non-infected HeLa cell lysates was conducted (Jagdeo et al., 2018) (Figure 1B). Additionally, two controls were included: (i) CVB3 infected cell lysates collected at 7 hpi served as a positive control; (ii) recombinant 2A or 3C with the catalytic site mutation (2A^mut^ or 3C^mut^) were incubated with HeLa cell lysates for 16 h, which served as negative controls. Figure 1B demonstrates that only protease 2A could cleave NUP98 to produce a ~43 kDa fragment, which is the same size as that produced by CVB3 infection. These data suggest that viral protease 2A but not 3C is responsible for the cleavage of NUP98 during CVB3 infection.

### CVB3 Infection Induces the Redistribution of NUP98 to Cytoplasmic Punctate Structures

**Figure 2:**
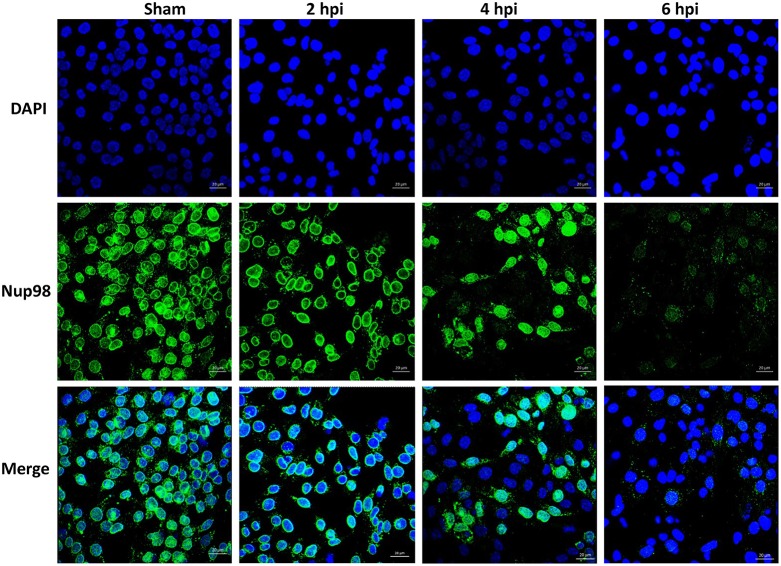
CVB3 infection induces the sub-cellular redistribution of NUP98 in cardiomyocytes. Mouse HL-1 cardiomyocytes were sham-infected with PBS or infected with CVB3 for 2–6 h. Cells were fixed and immunostained using a NUP98 specific antibody. Blue represents the nuclei stained with DAPI and green shows NUP98 protein. Beginning at 4 hpi, NUP98 was relocalized to the cytoplasm in punctate structures, becoming most apparent at 6 hpi. Cells were observed at each time-point by confocal microscopy at 20× magnification. Scale bars = 20 μm.

To examine whether CVB3-induced cleavage of NUP98 caused redistribution of NUP98 during infection, confocal microscopy was employed to observe its sub-cellular distribution after infection. HL-1 cardiomyocytes were infected with CVB3 at 10 MOI and fixed at 2, 4, and 6 hpi. Cells were immunostained with the primary NUP98 antibody, followed by Alexa 488-conjugated secondary antibody for confocal observation. As shown in Figure 2, NUP98 migrated from the nuclear periphery at 4 hpi into the nucleoplasm, where the signal was enhanced, likely due to cellular upregulation of NUP98 in response to CVB3 infection. At 6 hpi, NUP98 was observed in punctate structures mostly in the cytoplasm. Since we had verified that 2A was responsible for NUP98 cleavage, we wondered if the observed redistribution of NUP98 in punctate pattern could be triggered by expression of viral protease 2A alone.

### Ectopic Expression of Viral Protease 2A Is Sufficient to Induce the Sub-Cellular Redistribution of NUP98 to Punctate Cytoplasmic Structures

**Figure 3:**
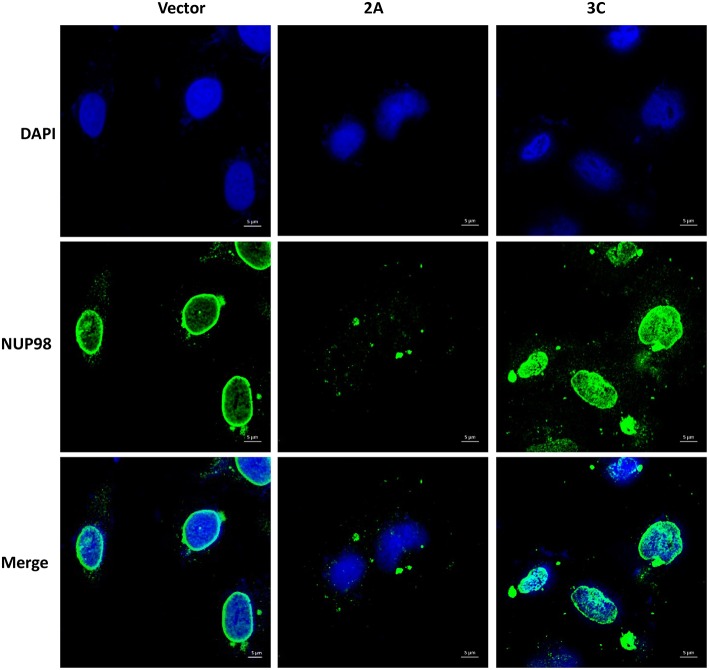
Ectopic expression of viral protease 2A or 3C results in differential sub-cellular redistribution of NUP98. HeLa cells were transfected with plasmid expressing viral protease 2A or 3C or with empty vector for 24 h. Cells were fixed and immunostained as described in Figure 2. Cells were visualized by confocal microscopy, images are shown at 60× magnification. Blue represents the nuclei stained with DAPI and green shows NUP98 protein. Scale bars = 5 μm. 2A transfected cells show NUP98 redistributed to the cytoplasm, indicating cleavage.

To determine if viral protease 2A alone could induce the subcellular redistribution of NUP98 during CVB3 infection, HeLa cells were transfected with a plasmid expressing the 2A (pIRES-2A), 3C (pIRES-3C), or empty vector (pIRES) for 48 h in the absence of CVB3 infection and subsequently fixed and immunostained for confocal microscopy examination. As demonstrated in Figure 3, ectopic expression of protease 2A induced the subcellular redistribution of NUP98 from the nuclear periphery to nucleoplasm and then to cytoplasm as compared to vector transfected cells where NUP98 remained localized to the nuclear periphery. This result suggests that 2A expression is sufficient to induce a similar accumulation and subcellular redistribution of NUP98 in punctate structures as seen at 6 h post-CVB3 infection in Figure 2. Intriguingly, ectopic expression of viral protease 3C in the absence of viral infection appeared to induce the upregulation and redistribution of NUP98 to the nucleoplasm, as compared to vector transfected cells where NUP98 is observed mostly at the nuclear periphery (see Discussion).

### siRNA Knockdown of NUP98 Differentially Regulates Cardioprotective and Viral Gene Expression While Overexpression of NUP98 Produces the Inverse Effect

Previous studies report that NUP98 acts as a transcription factor for NRG1 during human development (Liang et al., 2013). Additionally, NRG1 has been used in clinical trials for the treatment of myocardial infarction in which increased cardio-protective effects were observed. NRG1 binding to its receptor ERBB4 activates pathways which promote cardiac myocyte survival, sarcomeric organization, cell to cell contact and cardiac pumping (Xu et al., 2010).

**Figure 4:**
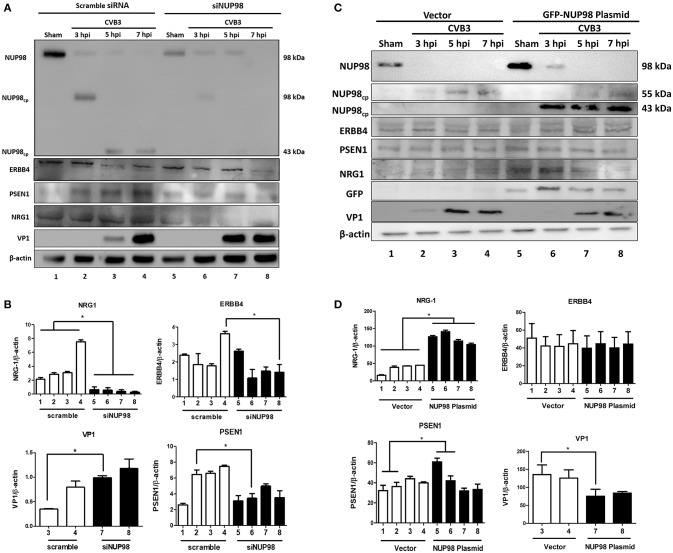
siRNA knockdown or overexpression of NUP98 differentially regulates NRG1, ERBB4, PSEN1, and VP-1 expression. **(A)** HeLa cells were transfected with scrambled or NUP98 siRNA for 48 h. Cells were subsequently infected with CVB3 or sham-infected with PBS. Cell lysates were harvested at the indicated time-points for western blot analysis using the indicated antibodies. β-actin was used as a loading control. **(B)** Quantification of protein expression. Densitometry was performed on the western blot images using the NIH ImageJ program. The data were normalized against the loading control (*n* = 3, *p < 0.05) and displayed graphically. Each scrambled siRNA transfected protein was compared to the corresponding timepoint of siNUP98 transfected cells. **(C)** HeLa cells were transfected with GFP-NUP98 plasmid or vector only for 48 h and then infected with CVB3 or sham-infected (PBS). Cell lystates were harvested at the indicated time-points for western blot analysis. **(D)** Quantification of protein expression. Densitometry and data normalization were performed as described for **(B)** (*n* = 3, *p < 0.05) and displayed graphically.

To examine the effect of NUP98 knockdown during CVB3 infection on the expression of NRG1, ERBB4, PSEN1 and viral capsid protein VP1 (a marker of viral protein synthesis), siRNA targeting NUP98 (siNUP98) or scrambled siRNA was transfected into HeLa cells 48 h prior to infection with CVB3 or sham-infection with PBS. Lysates were collected from sham-infected and CVB3-infected cells at 3, 5, and 7 hpi. Western blot analysis confirmed the successful knockdown of NUP98 by siRNA (Figure 4). Following a decrease in NUP98 levels after siNUP98 knockdown, NRG1 levels in sham-infected/scrambled siRNA-treated cells were ~3-fold higher than that in siNUP98 treated/sham-infected cells (Figures 4A,B, lane 1 vs. 5), indicating that NUP98 upregulates NRG1 in the absence of CVB3 infection. Interestingly, NRG1 expression levels during infection were significantly lower in siNUP98 treated cells than in scrambled siRNA treated cells (Figures 4A,B, lane 6–8 vs. 2–4), particularly at 7 hpi, the decrease is ~20-fold (lane 4 vs. 8). Other proteins including PSEN1 and ERBB4 were also decreased in siNUP98-treated cells compared to their controls; but CVB3 VP1 was significantly increased in siNUP98 treated cells compared to the scrambled siRNA treated control (lane 3 vs. 7). However, at 7 hpi, the VP1 level only had a slight increase, which is likely due to the complete cleavage of NUP98.

‘To observe the effect of NUP98 overexpression on its target gene during CVB3 infection, GFP-NUP98 plasmid or GFP vector was transfected into HeLa cells, infected with 10 MOI CVB3 and collected at 3, 5, 7 hpi. Following an increase in NUP98 levels, NRG1 protein expression increased in both sham- and CVB3-infected cells relative to vector transfected cells (Figures 4C,D, lane 5–6 vs. 1–2), confirming a regulatory role for NUP98 on NRG1 expression in presence and absence of CVB3 infection. CVB3 infection in NUP98 plasmid-transfected cells also decreased the resulting level of NRG1 at 5 and 7 hpi (after an initial increase in NRG1 levels at 3 hpi induced by NUP98 overexpression) (Figures 4C,D, lane 5–6 vs. 7–8). Although PSEN1 and ERBB4 expression only showed a slight change, the dramatic increase in NRG1 expression caused a significant decrease of VP1 level.

The significance of these data is 2-fold: first, CVB3 infection can reduce the NUP98-mediated up-regulation of cardio-protective NRG1, which is likely attributed to the cleavage of NUP98 by CVB3 protease 2A. Second, the siNUP98 treatment further enhances the CVB3-induced downregulation of NRG1 via silencing of NUP98, whereas overexpression of NUP98 has the opposite effect.

### siRNA Knockdown of NUP98 Enhances Viral Particle Formation and Decrease Cell Viability During Early Phase of Infection

**Figure 5:**
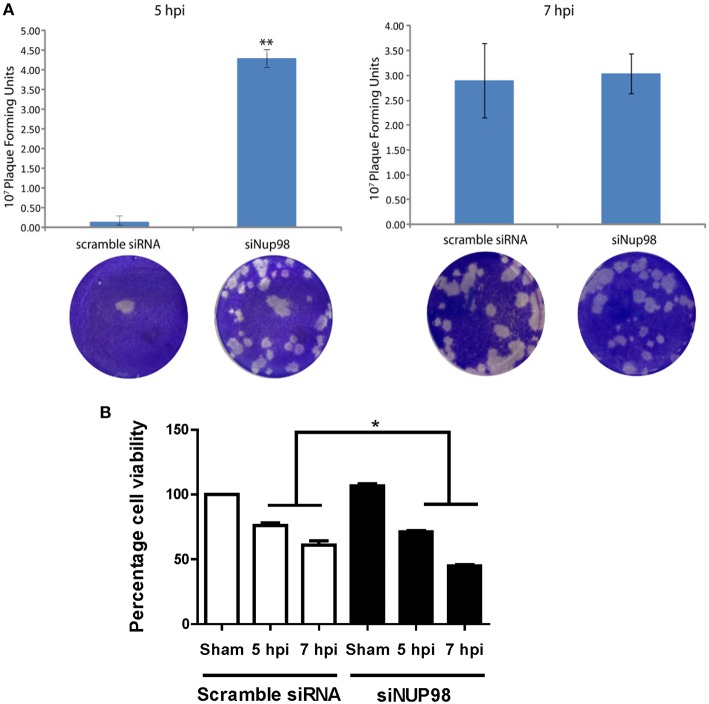
siRNA knockdown of NUP98 enhances viral particle formation and reduces cell viability. **(A)** HeLa cells were transfected with scrambled or NUP98 siRNA for 48 h. Cells were subsequently infected with CVB3 or sham-infected with PBS for 5 or 7 h. Supernatants were collected and used to measure viral particle release by viral plaque assay. Data were subjected to statistical analysis, *n* = 3, **p < 0.01. **(B)** Cell viability assay. HeLa cells were transfected with scrambled siRNA or siNUP98 for 48 h and then infected with CVB3 or sham-infected with PBS for 5 or 7 h. MTS viability assay was performed. Sham infected control cell viability was set to 100% survival and other data were converted to percentage of the control, *n* = 3, *p < 0.05.

To determine the effect of NUP98 knockdown on infectious viral particle formation, supernatants were collected from CVB3 infected HeLa cells transfected with scrambled siRNA or siNup98 for viral plaque assay as described in the Materials and Methods. The results show that at 5 hpi the Nup98 knockdown cells had ~20-fold higher viral titer at the 10^−7^ dilution than scrambled siRNA-treated control cells at the sham dilution, even though a slight increase of CVB3 particles was observed at 7 hpi (Figure 5A). To determine the effect of siRNA knockdown of NUP98 on cell viability against viral infection, MTS assay was performed in CVB3-infected HeLa cells transfected with scrambled siRNA or siNup98. The sham-infected cells were the control. At both 5 and 7 hpi, siNUP98 treated wells showed significantly lower cell viability than scrambled siRNA treated cells, while no significant difference in cell viability was found in siNUP98 vs. scrambled siRNA treated sham-infected cells (Figure 5B). These results directly support the antiviral role of NUP98, as Nup98 silencing in CVB3 infection results in increased cell death while this silencing produces no effect on cell survival in non-infected states.

### Nup98, Nrg1, and erbB4 Are Upregulated in the Acute Phase but Downregulated in Later Phase of CVB3 Infected Mouse Myocardium

**Figure 6:**
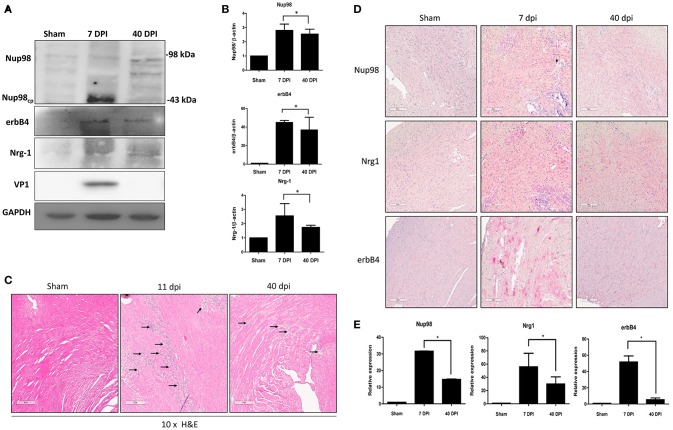
Nup98, Nrg1, and erbB4 are upregulated at early phase of infection and were downregulated at later phase in mice. Four-weeks-old A/J mice were infected with CVB3 at 10^5^ PFU or sham-infected with PBS. Mouse heart tissues were harvested at the indicated days post-infection. **(A)** Tissue lysates was subjected to western blot analysis of NUP98 cleavage products and its downstream gene expression using the indicated antibodies. **(B)** Quantification of protein expression levels. Densitometry was performed on western blot bands using the NIH ImageJ program as described in Figure 4B (*n* = 3, *p < 0.05). **(C)** H&E staining. Tissues were fixed in formalin and paraffin embedded at time of harvest corresponding to dpi. Images shown are at 10× magnification. Arrows indicate inflammatory infiltration. **(D)** Tissue was fixed in formalin and paraffin embedded. Immunohistochemistry was performed using the indicated antibodies. Red staining indicates the protein of interest. The Images were taken at 10× magnification. Scale bar = 200 μm. **(E)** Quantification of the expression levels of Nup98, Nrg1, and erbB4 was conducted using the Aperio ImageScope software. Expression for sham-infected control was set to 1 and relative expression for 7 and 40 dpi was normalized against the control, *n* = 3, *p < 0.05.

To verify our tissue culture findings in an *in vivo* setting, a mouse model of viral myocarditis was employed. Four-weeks old male A/J mice were infected with 10^4^ PFU of CVB3 or sham-infected with PBS. Hearts were harvested at 7 (acute infection phase) and 40 (chronic) dpi, corresponding to acute viral infection and innate immune response. Tissue was lysed for western blot analysis of Nup98 cleavage and its target gene expression, or formalin fixed/paraffin embedded and stained by immunohistochemistry for gene expression and myocarditis. The results showed that Nup98, Nrg1, and erbB4 were upregulated during the acute phase of infection (7 dpi) and decreased at the chronic phase of infection (40 dpi) even though the extent of decrease for Nup98 expression was small (Figures 6A,B). Note that at 40 dpi VP1 was non-detectable, implying the clearance of viral particles by host immune system after the viremic phase. Myocarditis started to occur after 6 dpi (data not shown) and reached a higher level at 11 dpi (Figure 6C). Immunohistochemistry demonstrated that Nup98, Nrg1 and erbB4 were all upregulated at 7 dpi and downregulated at 40 dpi, a time point of heart recovery from acute infection; however, at this time point their expression levels were still higher than that of the sham infected hearts (Figures 6D,E), indicating the strong immunoreactivity of proteins in the Nup98-Nrg1-erbB4 signaling axis during acute viral myocarditis *in vivo*.

## Discussion

The dual functional roles of NUP98 in serving as a nuclear “gatekeeper” and an immune-responsive transcription factor are well-documented (Iwamoto et al., 2010; Panda et al., 2014). NRG1 is a direct transcriptional target gene of NUP98 and functions along with its receptor ERBB4 in various normal and pathological conditions including cardiac development, cancer and neurodegeneration diseases, etc. (Xu et al., 2010; Simon and Rout, 2014). In addition, PSEN1, a cellular protease responsible for processing the tyrosine kinase receptor ERBB4, plays an important role in the NRG1-ERBB4 signaling cascade. Our studies here provide evidence to demonstrate coordination between NUP98 and NRG1-ERBB4/PSEN1 signaling proteins in the host response to viral infection and identify a previously unrecognized mechanism of enteroviral pathogenesis of viral myocarditis. Firstly, we found that NUP98 was cleaved in HeLa cells during CVB3 infection to produce a ~55 kDa fragment at as early as 3 hpi. This cleavage further progressed to produce a ~43 kDa product at 4 hpi, indicating that NUP98 protein was cleaved at two sites during CVB3 infection. This cleavage pattern is similar to that of 2A of poliovirus and human rhinovirus although the sizes of their cleavage products are slightly different (Park et al., 2015). To further determine which protease(s) caused these cleavages, we performed *in vitro* cleavage assays using purified recombinant CVB3 2A and 3C proteases. Only 2A was able to generate the same cleavage pattern as that produced by CVB3 infection, suggesting that CVB3 2A but not 3C is responsible for the cleavage of NUP98 protein. Intriguingly, we found that 2A cleavage during infection corresponded with increased CVB3 VP1 protein production. This implies a connection between increased NUP98 cleavage and increased viral replication; intact NUP98 can potentially suppress CVB3 replication through its immune-responsive transcription activity. This finding is in line with the result obtained from studies in infection of Drosophila by human arboviruses (Panda et al., 2014).

Disruption of the nuclear pore complex during viral infections resulting in loss of nuclear membrane integrity and attenuation of nucleo-cytoplasmic trafficking has been previously described (Levin et al., 2014; Watters et al., 2017), occurring through proteolytic cleavage of NUPs, perturbing the balance of molecules (i.e., RNA and protein) available between the nucleus and cytoplasm. In the cytoplasm, attenuation of this trafficking will block nuclear import of proteins, thus leading to accumulation of certain nuclear proteins, such as the IRES-trans activation factors (ITAF) La, PTB, Sam68, etc., in the cytosol (Flather and Semler, 2015). Conversely, cleavage of NUPs will increase permeability of the nuclear membrane, resulting in leaking of nuclear resident ITAFs to cytoplasm (Belov et al., 2004). The increased availability of ITAFs in cytosol may enhance the IRES-driven translation of CVB3 genome because its genome harbors an IRES in its 5′UTR. On the other hand, for the nucleus side, the blockage of nucleo-cytoplasmic communication will suppress the nuclear export of mRNAs encoding proteins involved in host antiviral immune responses. This speculation is supported by previous findings in several picornavirus studies in which the expression of proinflammatory cytokines and chemokines as well as antiviral transcription factors are suppressed due to the shortage of their mRNAs in cytoplasm (Flather and Semler, 2015; Lloyd, 2015). All these processes benefit viral replication and are harmful to cell survival.

Clearly NUP98 cleavage by CVB3 2A plays an important role in viral pathogenesis. However, we cannot ignore the role of other partners in this type of mechanism since 2A protease has multiple cleavage substrates, such as eIF4GI, eIF4GII, PABP, etc.; cleavage of these proteins can shut down host cap-dependent translation initiation and in turn promote cap-independent, IRES-driven translation of enteroviruses (Etchison et al., 1982; Gradi et al., 1998; Kuyumcu-Martinez et al., 2004). Thus, the protease-mediated viral pathogenesis is a combined effect of cleavage of multiple substrates. However, it is worth mentioning that NUP98 is an interferon-inducible transcription factor and plays a critical role in host immune response against viral infection (Panda et al., 2014). In addition, NUP98 is very sensitive to viral protease and can be cleaved by 2A more rapidly and earlier than eIF4GI cleavage (Park et al., 2008); therefore NUP98 is one of the cleavage targets that play a major role in viral pathogenesis compared to other substrates.

In the immune staining for NUP98 subcellular localization, we found that CVB3 2A-induced NUP98 cleavage elicited further translocation of NUP98 fragments to the nucleoplasm at early time point (2 hpi) and subsequently to the cytoplasm in a punctate pattern at later times (Figure 3). This observation is similar to what has seen in poliovirus infection (Castello et al., 2009). This feature may be caused by the gradual destruction of nuclear membrane by viral protease 2A and/or other proteases. The punctated pattern of NUP98 distribution may represent the host cell response to viral insult. Although the role of these structures during CVB3 infection has yet to be identified, it is possible that NUP98 localizes to P-bodies, stress granules and/or vesicles for the protection of cells from viral invasion. This speculation is supported by similar redistribution patterns induced by other viral infections, such as poliovirus and human rhinovirus (Lloyd, 2016). In fact, two recent studies have demonstrated that NUP98 protein is indeed present in stress granules produced during cellular stress conditions induced by non-infection conditions (Jain et al., 2016; Zhang et al., 2018). Interestingly, expression of viral protease 3C also induced redistribution of NUP98 to the nucleoplasm, despite no detectable protease effect of 3C on NUP98 was observed (Figure 3). The likely mechanisms for this could be that 3C expression causes cleavage of other unknown substrates of the NUP complex, but this cleavage is not sufficient for destruction of the nuclear membrane. Thus, NUP98 is limited in the nucleoplasm without relocation to cytoplasm. This speculation is indirectly supported by studies of human rhinovirus, which demonstrated that human rhinovirus 3C cleaves NUP153, leading to the misallocation of NUPs in infected host cells (Walker et al., 2013). Whether CVB3 3C can cleave NUP153 and other NUPs needs to be investigated in the future.

To further verify the role of NUP98 in regulating target gene expression and antiviral effect during infection, siRNA targeting NUP98 was applied to silence its expression. These data show that knockdown of NUP98 during CVB3 infection further promoted viral replication, which was evidenced by the further increase of CVB3 VP1 synthesis and viral progeny production compared to scrambled siRNA-treated cells at the 5 hpi. However, by 7 hpi the VP1 production in both the siNUP98-treated and scrambled control siRNA-treated cells reached similarly high levels, and these levels are significantly higher than that at 5 hpi in control siRNA-treated cells (Figure 4). At this later time point, VP1 production in cells with NUP98 knockdown returned to the same level as that of its control cells, implying knockdown treatment has no effect anymore on NUP98-mediated regulation of VP1 production as NUP98 could be completely cleaved. Indeed, at 7 hpi the full-length NUP98 was almost completely absent. Overall, these data support the notion that NUP98 acts as an antiviral transcription factor during CVB3 infection.

Furthermore, NRG1 and PSEN1, known for their roles in cardioprotection (Liu et al., 2006; Li et al., 2011; Galindo et al., 2014), were further decreased compared to cells treated with control siRNA. However, ERBB4, the receptor of NRG1, had a slight decrease at early time-point of infection and decreased dramatically at 7 hpi. This result suggests that the genes NRG1 and PSEN1, as the downstream targets of NUP98, play a positive role with the assistance of ERBB4 in NUP98-mediated suppression of viral infection and improvement of host cell viability. This finding on NUP98's antiviral effect was further substantiated by ectopic expression of NUP98, showing the decreased CVB3 replication upon overexpression of NUP98. These *in vitro* data were further substantiated in *in vivo* experiments. Employing a well-established murine model of viral myocarditis, we showed that Nup98, Nrg1, and erbB4 are all upregulated in the myocardium during the acute phase (7 dpi). However, at a later chronic phase of infection (40 dpi), expression of all three were downregulated. This reduced expression of cardioprotective genes may contribute to pathogenesis of the disease. Intriguingly, the expression pattern of proteins in the Nup98-Nrg1-erbB4 signaling cascade is not only limited in infected region but is enhanced in regions with no inflammation or apparent tissue damage, suggesting a pathologic field effect. Further studies are needed to confirm these findings; however, it is worth mentioning that this finding is valuable because it may provide sensitive diagnostic markers for human viral myocarditis as they are expressed at high levels during the early phase of infection.

Taken together, this study demonstrated that NUP98, an important regulator of antiviral gene transcription, is targeted by coxsackievirus protease 2A early on in the model of CVB3 myocarditis. Moreover, we found that cleavage of NUP98 leads to the downregulation of cardioprotective genes Nrg1 and erbB4 in later phase of infection. This finding may provide evidence to explain, at least in part, why CVB3 infection causes target cell damage in the heart. In addition, the immune histopathology studies revealed the timing, location and expression levels of these proteins involved in the Nup98-Nrg1-erbB4 signal cascade. These data are not only valuable information for adding new diagnostic markers, but also may provide potential drug targets for developing treatments. To our knowledge this is the first study linking NUP98 cleavage and its downstream target gene expression to the molecular pathogenesis of viral myocarditis.

## Data Availability

The raw data supporting the conclusions of this manuscript will be made available by the authors, without undue reservation, to any qualified researcher.

## Ethics Statement

This study was carried out in strict accordance with the recommendations in the Guide to the Care and Use of Experimental Animals—Canadian Council on Animal Care. All protocols were approved by the Animal Care Committee of Faculty of Medicine, University of British Columbia (protocol number: A16-0093).

## Author Contributions

PH, DY, and DG conceived and designed the experiments. PH, AH, YQ, HZ, GZ, CL, SS, VL, VC, and GF performed the experiments. PH, DY, DG, VC, and BM wrote the manuscript. MV and EJ provided reagents.

### Conflict of Interest Statement

The authors declare that the research was conducted in the absence of any commercial or financial relationships that could be construed as a potential conflict of interest.
